# Multicomponent parenteral lipid emulsions do not prevent liver injury in neonatal pigs with obstructive cholestasis

**DOI:** 10.1172/jci.insight.189196

**Published:** 2025-04-17

**Authors:** Greg Guthrie, Caitlin Vonderohe, Valeria Meléndez Hebib, Barbara Stoll, Douglas Burrin

**Affiliations:** 1 USDA-ARS Children’s Nutrition Research Center, Department of Pediatrics, Baylor College of Medicine, Houston, USA.; 2 USDA-ARS Children’s Nutrition Research Center, Department of Pediatrics, Section Gastroenterology, Hepatology and Nutrition, Baylor College of Medicine, Houston, USA.

**Keywords:** Hepatology, Metabolism, Fibrosis

## Abstract

Biliary atresia (BA) is a pediatric liver disease that often necessitates parenteral nutrition (PN) to support growth due to impaired liver function. While soy-based lipid emulsions (SLE) are commonly used in PN, they may contribute to cholestatic liver injury. In contrast, mixed lipid emulsions (MLE) show promise in preventing cholestasis in infants without BA, potentially by restoring bile flow. However, their effectiveness in patients of complete bile duct obstruction, as seen in BA, remains uncertain. To explore the potential benefits of MLE in BA, we utilized a neonatal pig model of bile duct ligation (BDL). Pigs underwent either BDL or sham surgery and were subsequently fed either MLE or SLE via PN, or enterally with formula. The MLE-BDL pigs exhibited significantly greater weight gain compared with those fed SLE or formula enterally. Additionally, MLE-BDL pigs showed higher serum bile acid and γ-glutamyl transferase concentrations compared with SLE-BDL pigs. However, no significant differences in liver injury, assessed by ductular reaction or fibrosis, were observed between MLE- and SLE-BDL pigs. Based on weight gain alone, MLE may be a superior lipid emulsion for use in neonates with obstructive cholestasis.

## Abstract 2

Multicomponent lipid emulsions enhance weight gain in obstructive cholestasis compared to enteral or soy-based parenteral nutrition, but do not protect against liver injury.

## Introduction

Neonatal cholestasis arises from the impairment of bile flow or reduced clearance of bile. The components of bile, notably bilirubin and bile acids, accumulate, leading to hepatic inflammation and injury. The most common form of obstructive neonatal cholestatic liver disease, biliary atresia (BA), occurs in 1 in 12,000 live births in the United States (1) and varies between 0.5 and 1.6 in 10,000 live births globally (2, 3). BA is a fibro-obliterative cholangiopathy, which causes complete loss of extrahepatic bile ducts in most patients. Infants diagnosed with BA undergo surgical Roux-en-Y anastomosis called the Kasai procedure (hepatoportoenterostomy) to restore bile flow from the liver to the intestines. However, approximately 30%–50% of infants who receive a hepatoportoenterostomy do not regain adequate bile flow and require liver transplant within 2 years of life (4). Infants with BA have multiple challenges to meet energy requirements due to impaired fat absorption and increased metabolic stress (5). The inability to meet these energy requirements can result in failure to thrive, which is associated with need of early transplantation and death at 2 years (6). Thus, in infants with BA with either failed or inadequate enteral (ENT) feeding, parenteral nutrition (PN) becomes a necessary intervention to maintain the growth needs of this at-risk population.

PN provides essential nutrients and energy for many infants, supporting their growth and survival (7, 8). However, prolonged PN use (>2 weeks) can lead to PN-associated liver disease (PNALD) (9), which is characterized by elevated serum markers (direct bilirubin, bile acids, transaminases), along with liver damage, immune cell infiltration, and fibrosis (9, 10). While all components of PN have been investigated for their potential role in causing PNALD, the lipid component has been the most frequently identified as a direct contributor (11, 12). Soy-based lipid emulsions (SLE) were once the primary choice for PN, but concerns about their potential role in the development of PNALD have led to increased adoption of fish oil and mixed lipid emulsions (MLE) as potentially safer alternatives (13–18).

While there has not been a clear consensus on what component in SLEs drive PNALD development, some factors have been postulated. SLEs are high in omega-6 fatty acids and low in omega-3 fatty acids, which may promote the production of proinflammatory mediators (19–21). They also contain phytosterols, cholesterol-like molecules produced exclusively in plants that may antagonize bile acid homeostasis (22) and promote an inflammatory response (23–25). To address this issue, new generation MLEs (SMOFlipid) have been developed containing soy oil, fish oil, olive oil, and medium chain triglycerides (26). MLEs have higher concentrations of omega-3 fatty acids, primarily docosahexaenoic acid (DHA) and eicosapentaenoic acid (EPA), and have lower phytosterol content. While there have been conflicting clinical reports, some clinical studies support the use of MLEs to prevent or resolve cholestasis during PN (27–31). While many groups suggest that the potential benefit of MLEs comes from the better balanced fatty acid composition and reduced phytosterols concentration, recent research from our lab suggests that MLEs maintain normal bile flow into the gut, which could explain why it prevents cholestasis or PNALD (32). Thus, it is important to establish whether MLEs are hepatoprotective in circumstances when patients are given PN, but bile flow is obstructed, as is the case with obstructive cholestatic liver diseases.

From the limited data available, the use of SLE in PN during BA is effective in maintaining growth and body weight, yet liver disease can be more severe than ENT feedings (33). It is clinically relevant to know if MLE administration in BA is appropriate to both support growth and reduce the progression of liver injury compared with current SLEs. A recent retrospective study on the use of PN during BA by Wendel et al. did not show accelerated liver disease in patients receiving PN, but they did not include the lipid source and likely have fish oil–based emulsions in their cohort (34). In this study, we will provide a direct test of whether MLE provides hepatoprotection and supports normal growth compared with SLE in neonatal PN-fed pigs with obstructive cholestasis. The results from this study will have direct implications for the choice of lipid emulsion in infants with BA who need PN support.

## Results

### MLE does not improve serum chemistry markers of cholestasis or liver injury.

**Figure 1:**
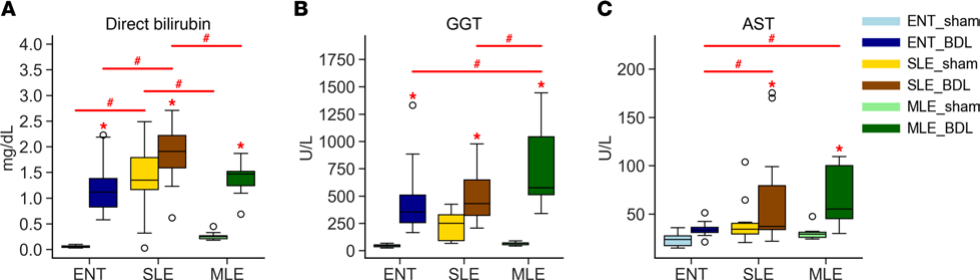
Serum chemistry markers are increased in all groups with BDL. (**A**–**C**) Direct bilirubin is a marker of cholestasis, γ-glutamyl transferase (GGT) is a marker of cholangiocyte injury, and aspartate amino transferase (AST) is a marker of hepatocyte injury. Statistical significance for box plots was determined via 2-way ANOVA and Tukey’s post hoc comparison. *P < 0.05, from within-diet comparisons, and ^#^*P* < 0.05 from within-surgery treatments. Box plots lines represent quartiles, whiskers represent largest value within 1.5× interquartile range, and open circles represent outliers. *n* = 9–12/group.

Serum direct bilirubin is a standard marker used to determine cholestasis in clinical settings. In all groups with bile duct ligation (BDL), direct bilirubin was significantly elevated compared with their diet controls (Figure 1A). Within the BDL groups, SLE-BDL direct bilirubin was significantly higher than both ENT-BDL (ENT-BDL) and MLE-BDL. The BDL groups also exhibited increased serum concentrations of markers of hepatic injury. Serum γ-glutamyl transferase (GGT), a marker of cholangiocyte injury often seen elevated in obstructive cholestasis in infants, was significantly elevated in all BDL groups compared with their sham controls (Figure 1B). The MLE-BDL had the largest increase in serum GGT, which was significantly greater than both ENT-BDL and SLE-BDL. The hepatocyte marker of injury, aspartate aminotransferase (AST), was significantly elevated in the TPN groups that had BDL (Figure 1C). However, the ENT-BDL group AST concentration was identical to that of the ENT-sham. While albumin (Supplemental Figure 1A; supplemental material available online with this article; https://doi.org/10.1172/jci.insight.189196DS1) remained consistent, globulin (Supplemental Figure 1K) increased significantly in both ENT-BDL and MLE-BDL, relative to their respective sham groups. This led to a significant decrease in the albumin/globulin (Supplemental Figure 1B) ratio only in the ENT-BDL group.

### MLE improves growth rate during obstructive cholestasis.

**Figure 2:**
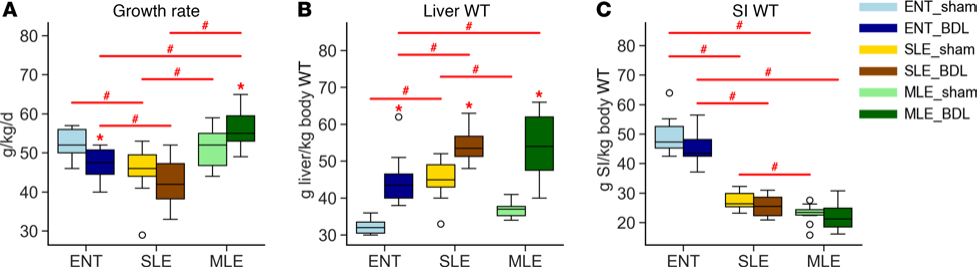
MLE diet leads to greater weight gain during BDL. (**A**) Weight gain was measured every other day in pigs and their growth rate was calculated as grams / (kg * day). (**B** and **C**) Liver weight small intestinal (SI) weight were expressed as gram tissue weight per kg body weight. Statistical significance for box plots was determined via 2-way ANOVA and Tukey’s post hoc comparison. *P < 0.05 from within-diet comparisons, and ^#^*P* < 0.05 from within-surgery treatments. Box plots lines represent quartiles, whiskers represent largest value within 1.5× interquartile range, and open circles represent outliers. *n* = 9–12/group.

Infants with BA are often at risk of failure-to-thrive due to poor absorption of dietary fats, and this is compounded by metabolic dysfunction secondary to liver injury. In our pig model, ENT-BDL pigs also had a significantly impaired growth rate compared with ENT-sham pigs (Figure 2A). The SLE-sham and SLE-BDL pigs were not significantly different from each other but overall had poorer growth compared with ENT or MLE pigs. Surprisingly, the MLE-BDL pigs had a comparable growth rate to ENT-sham and made up the only BDL group with an increase in growth rate compared with their sham control. In contrast to growth rate, liver weight significantly increased for all BDL groups compared with sham controls (Figure 2B). Within the BDL groups, TPN feeding led to significantly larger livers in the SLE-BDL and MLE-BDL groups compared with the ENT-BDL groups, yet liver weight did not differ between SLE-BDL and MLE-BDL. The combination of BDL and TPN did not affect the growth of the intestines, but consistent with other TPN studies (35, 36), groups that received PN (SLE and MLE) had small intestines that were about 50% the weight of ENT diet pigs (Figure 2C).

### MLE does not reduce liver injury during obstructive cholestasis.

**Figure 3:**
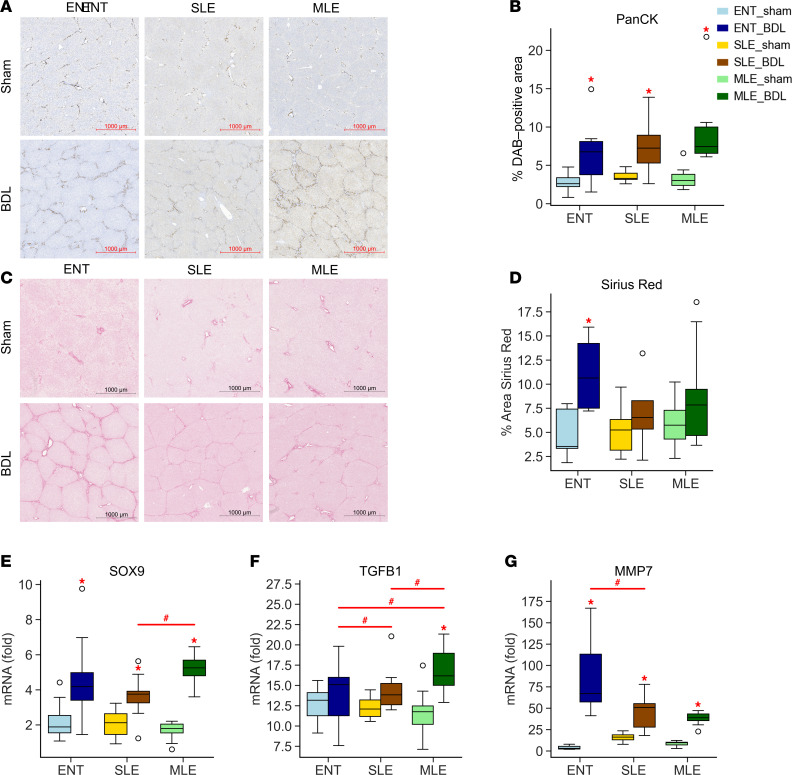
BDL promotes liver injury within all diet groups. (**A** and **B**) Ductular reaction was assessed with pancytokeratin staining and quantified using DAB-positive area (%) stained within a 9 mm^2^ field of view (5 fields per slide). (**C** and **D**) Collagen deposition and fibrosis were assessed using Sirius red stained liver slides and quantified using positive area (%) stained within a 9 mm^2^ field of view (5 fields per slide). (**E**–**G**) Real-time PCR was used to examine gene targets of ductular reaction SRY-Box Transcription Factor 9 (Sox9) (**E**), transforming growth factor beta (Tgfβ) (**F**), and matrix metalloproteinase 7 (Mmp7) (**G**). Statistical significance for box plots was determined via 2-way ANOVA and Tukey’s post hoc comparison. *P < 0.05 from within-diet comparisons, and ^#^*P* < 0.05 from within-surgery treatments. Box plots lines represent quartiles, whiskers represent largest value within 1.5× interquartile range, and open circles represent outliers. *n* = 4-–12/group.

We evaluated liver histopathology to identify hepatocellular injury resulting from BDL. Ductular reaction occurs in many forms of obstructive cholestasis in response to cholangiocyte and hepatocyte injury. In the pigs with BDL, there was a significant increase in the number of pancytokeratin-stained (panCK-stained) cells (Figure 3, A and B). We also examined fibrosis, a manifestation of liver injury, by using Sirius red to stain collagen within the tissue (Figure 3C). The ENT-BDL pigs showed significant increases in collagen deposition compared with the ENT-sham group (Figure 3, C and D). In contrast, the TPN groups (SLE and MLE) had less collagen-positive staining and did not differ from their respective sham groups.

Furthermore, we investigated genes associated with the progression of liver injury (Figure 3, E–G). *SOX9*, a gene implicated in ductular reaction (37, 38), was significantly upregulated in all BDL groups compared with their respective sham controls. Several markers of fibrosis also increased in the BDL-treated pigs. *MMP7* mRNA significantly increased in all the BDL surgery groups compared with respective sham controls. The expression on ENT-BDL was the highest among the BDL groups and significantly different than SLE-BDL. *TGFB1* mRNA expression only increased in the MLE-BDL group, which was significantly different from both MLE-sham and the ENT-BDL and SLE-BDL groups.

### Bile acid clearance and composition differs between enteral and parenteral BDL groups.

**Figure 4:**
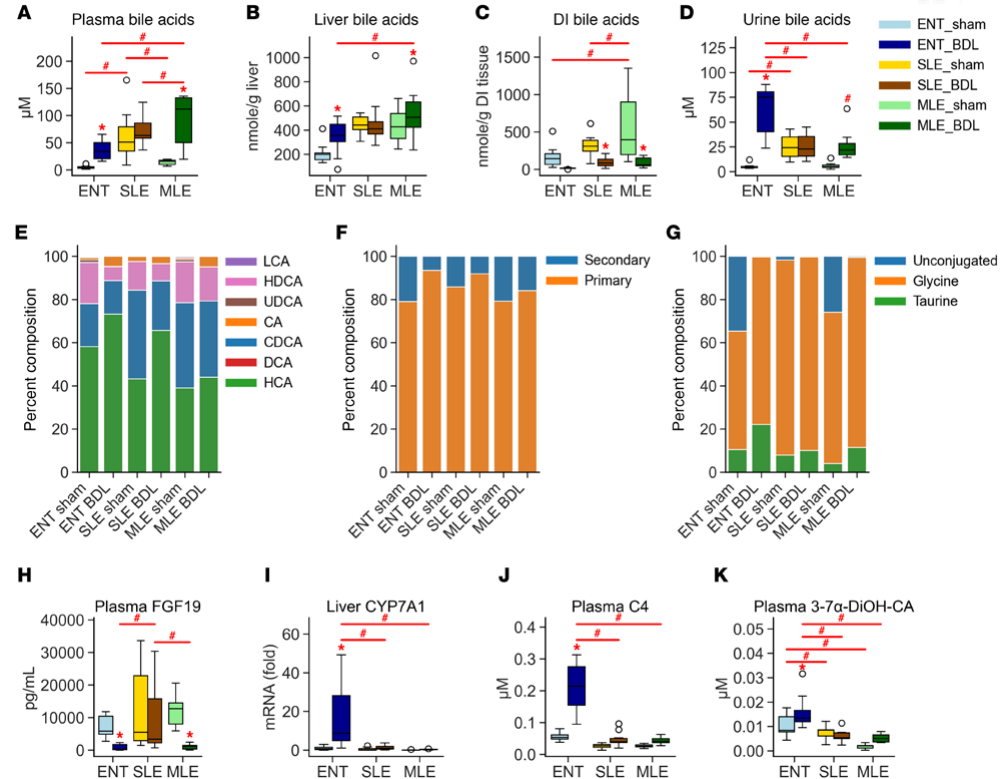
Bile acid pools, compositions, and regulatory pathways shift in response to BDL and feeding source. (**A**–**D**) The concentration of bile acids in 4 major compartments of bile circulation plasma, liver tissue, distal ileum tissue, and urine. (**E**–**G**) The composition of bile acids in the plasma broken down by bile acid (unconjugated + conjugated) (**E**), liver synthesized (PrimaryBA) or bacterial synthesized (SecondaryBA) (**F**), and conjugation status (**G**). (**H** and **I**) Regulators of bile acid synthesis were determined by ELISA plasma FGF19 and real time PCR cytochrome P450 7A1 (Cyp7a1). (**J** and **K**) Plasma concentration key intermediates of bile acid synthesis in the classical pathway plasma 7-α-Hydroxy-4-cholesten-3-one (C4, 7α-hydroxycholesterol) (**J**) and alternative pathway 3-7α-DiOH-CA (**K**). Statistical significance for box plots was determined via 2-way ANOVA and Tukey’s post hoc comparison. *P < 0.05 from within-diet comparisons, and ^#^*P* < 0.05 from within-surgery treatments. Box plots lines represent quartiles, whiskers represent largest value within 1.5× interquartile range, and open circles represent outliers. *n* = 5–12/group. LCA, Lithocholic acid; HDCA, hyodeoxycholic acid; UDCA, ursodeoxycholic acid; CA, cholic acid; CDCA, chenodeoxycholic acid; CDA, deoxycholic acid; HCA, hyocholic acid.

To understand the effect of BDL on bile acid homeostasis, we first examined the total bile acid concentration in 4 important compartments related to bile acid circulation: plasma, liver, distal ileum, and urine (Figure 4, A–D). Total plasma bile acids significantly increased in the ENT-BDL and MLE-BDL compared with their respective controls. Plasma bile acid levels in SLE-BDL did not differ from SLE-sham, as both the SLE-treated groups had high concentrations of bile acids (approximately 70 μM). The MLE-BDL had the highest concentration of plasma bile acids; they were significantly greater than both the ENT-BDL and SLE-BDL concentrations. Liver bile acid concentrations were elevated in ENT-BDL and MLE-BDL groups compared with their respective sham controls. Again, the MLE-BDL group had significantly higher hepatic bile acid concentrations compared with the ENT-BDL group. In contrast, the loss of bile flow from BDL led to significantly lower bile acid levels in the distal ileum of the SLE-BDL and MLE-BDL groups compared with their sham controls. Interestingly, the MLE-sham group had the highest concentration of bile acids in the intestine, more than 3 times that of the ENT-sham pigs. Bile acid levels were assessed in urine to quantify kidney clearance of plasma bile acid. ENT-BDL pigs had significantly more urinary bile content than ENT-sham, as well as SLE-BDL and MLE-BDL. The SLE-sham, SLE-BDL, and MLE-BDL urinary bile acid concentrations were the same. MLE-BDL urinary bile acid concentrations were significantly higher than MLE-sham concentrations.

In addition to the observed change in the compartmentalization of bile acids, we were also interested in whether the composition of bile acids shift after BDL and with differing dietary lipid source. We first examined the composition of individual bile acids that were pooled for their unconjugated and conjugated forms (Figure 4E). There was a large increase (26%) in the relative concentration of hyocholic acid (HCA) in the ENT-BDL compared with the ENT-sham and a large decrease (66%) in the concentration of hyodeoxycholic acid (HDCA). We observed similar increases (34%) in HCA in the SLE-BDL group compared with SLE-sham. However, HDCA did not decrease as much (40%) in the SLE-BDL versus SLE-sham comparison. The smallest change in relative bile acid composition occurred in the MLE-BDL versus MLE-sham comparison with a modest increase in HCA by 11%. We postulate that, by cutting off the flow of bile to the intestine with BDL, fewer primary bile acids are available to bacteria for conversion to secondary bile acids, therefore limiting the production of secondary bile acids observed in the BDL pigs. In Figure 4F, we examined the ratio of primary/secondary bile acid in each group to see if BDL ligation significantly reduced secondary bile acid relative concentrations. Surprisingly, all BDL groups still produced some secondary bile acids; however, the reduction in secondary bile acids was greatest in the ENT-BDL versus ENT-sham groups (69%). Additionally, we looked at the change in conjugated versus unconjugated bile acids, as unconjugated bile acids are likely to come from enterohepatic circulation returning to the liver (Figure 4G). Both ENT-sham and MLE-sham groups had unconjugated bile acids in the plasma. In contrast, plasma from SLE-sham only had about 1% of unconjugated bile acids. With BDL, both the ENT and MLE lost most of the unconjugated bile acids in circulation, with very little change in the conjugated and unconjugated composition in the SLE-BDL group. To further examine how bile acid homeostasis is regulated in obstructive cholestasis and PN, we examined the enterokine fibroblast growth factor 19 (*FGF19*), which negatively regulates the classic pathway of bile acid synthesis. Both the ENT-BDL and MLE-BDL groups had lower plasma FGF19 concentration compared with their respective sham controls (Figure 4H). Interestingly, SLE-BDL FGF19 concentrations were nearly identical to those of SLE-sham pigs. We then looked at the downstream target of FGF19, hepatic cytochrome P450 family 7 subfamily A member 1 (*CYP7A1*). In normal conditions, FGF19 suppresses the expression of *CYP7A1* mRNA, the rate-limiting enzyme in bile acid synthesis. As expected, the decrease in FGF19 in the ENT-BDL pigs led to a significant increase in *CYP7A1* mRNA expression (Figure 4I). However, in the MLE-BDL pigs, the decrease in FGF19 did not correlate to an increase in *CYP7A1* mRNA expression. In pigs administered with PN, the expression of *CYP7A1* mRNA was more than 20-fold lower compared with ENT-BDL pigs. CYP7A1 converts cholesterol to bile acids, producing hydroxysterol 7-α-Hydroxy-4-cholesten-3-one (C4) as a byproduct. In all groups, C4 levels mirrored the expression pattern of *CYP7A1* mRNA (Figure 4J). However, CYP7A1 regulates only the classical pathway of bile acid synthesis, not the alternative pathway. The concentration of the final product in this pathway, CA24-Δ5–3β,3-7-α-DiOH-CA, prior to its conversion to CDCA, was significantly elevated in ENT-BDL pigs compared with ENT-sham pigs (Figure 4K). There was no difference in this concentration between sham and BDL groups for either SLE or MLE diets.

### RNA-Seq analysis reveals shared inflammatory pathways and altered xenobiotic metabolism.

**Figure 5:**
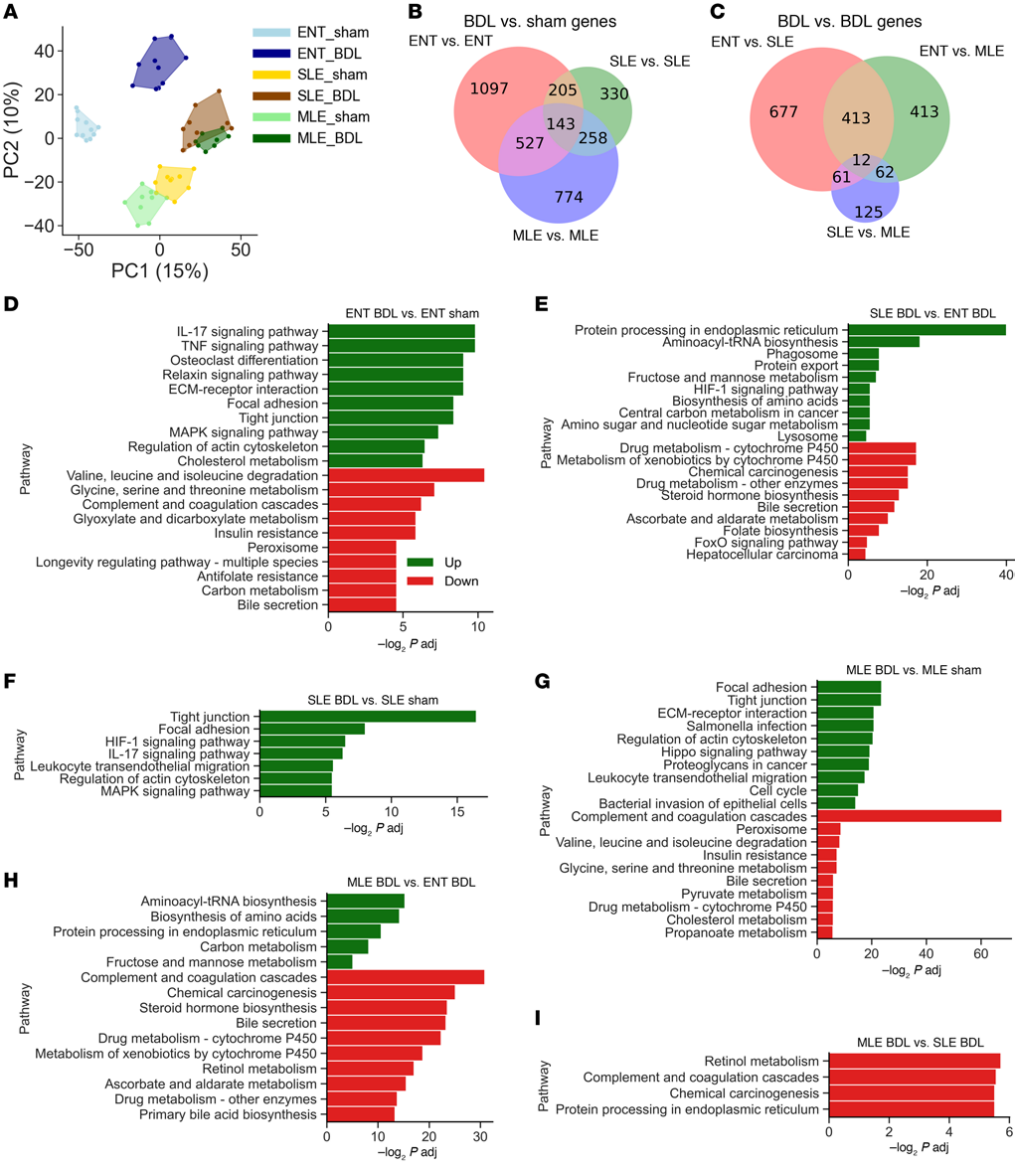
RNA-Seq analysis identifies genes’ signature changes in xenobiotic metabolism, circadian clock, bile acid metabolism, and inflammation. These are exacerbated by parenteral feeding. (**A**) Principle component analysis (PCA) of treatment groups. (**B** and **C**) Venn diagram of overlapping significantly altered genes (fold change > 2 and *q* < 0.05) for comparisons within-diet group (**B**) and between-diet groups (**C**) with BDL surgery. (**D**–**I**) Top 10 significantly upregulated (green) and downregulated (red) KEGG pathways for within-diet group (**D**–**F**) and between-diet groups (**G**–**I**) with BDL surgery. Pathway significance was determined using Fisher’s exact test. *n* = 7–12/group.

To understand the global hepatic gene expression changes in pigs after BDL and exposure to different lipid sources, we conducted RNA-Seq analysis of liver tissue. Principal component analysis (PCA) (Figure 5A) revealed distinct spatial separation between ENT-sham and ENT-BDL groups, as well as between PN-fed groups, regardless of sham or BDL treatment. ENT-BDL clustered uniquely from all other groups, while PN groups showed closer clustering but still separated by sham and BDL treatments.

Differentially expressed gene comparisons within groups (Figure 5B) indicated that ENT-sham versus ENT-BDL had the highest number of differentially expressed genes (1,097 genes), followed by MLE (724 genes), and SLE (330 genes). Only 143 differentially expressed genes were shared among all 3 diet groups. In comparisons between BDL groups (Figure 5C), the ENT versus SLE comparison had the most differentially expressed genes (677 genes), closely followed by ENT versus MLE (413 genes). The SLE versus MLE comparison resulted in 125 differentially expressed genes, with only 12 genes commonly changed among these groups.

Enriched Kyoto Encyclopedia of Genes and Genomes (KEGG) pathway comparisons within groups (Figure 5, D, F, and H) showed that inflammation and tissue remodeling pathways were predominantly upregulated across all diets. Amino acid and lipid metabolic pathways were significantly downregulated in the ENT and MLE groups, with no significant downregulation observed in the SLE group. In BDL surgery groups (Figure 5, E, G, and I), PN groups exhibited elevated expression of amino acid metabolism pathways compared with the ENT-BDL group. Downregulation was observed in pathways related to drug metabolism (P450 pathways) and bile metabolism and secretion. There were minimal differences between MLE-BDL and SLE-BDL; MLE-BDL livers showed no upregulated KEGG pathways, while downregulated pathways were related to vitamin A metabolism and clotting function.

**Figure 6:**
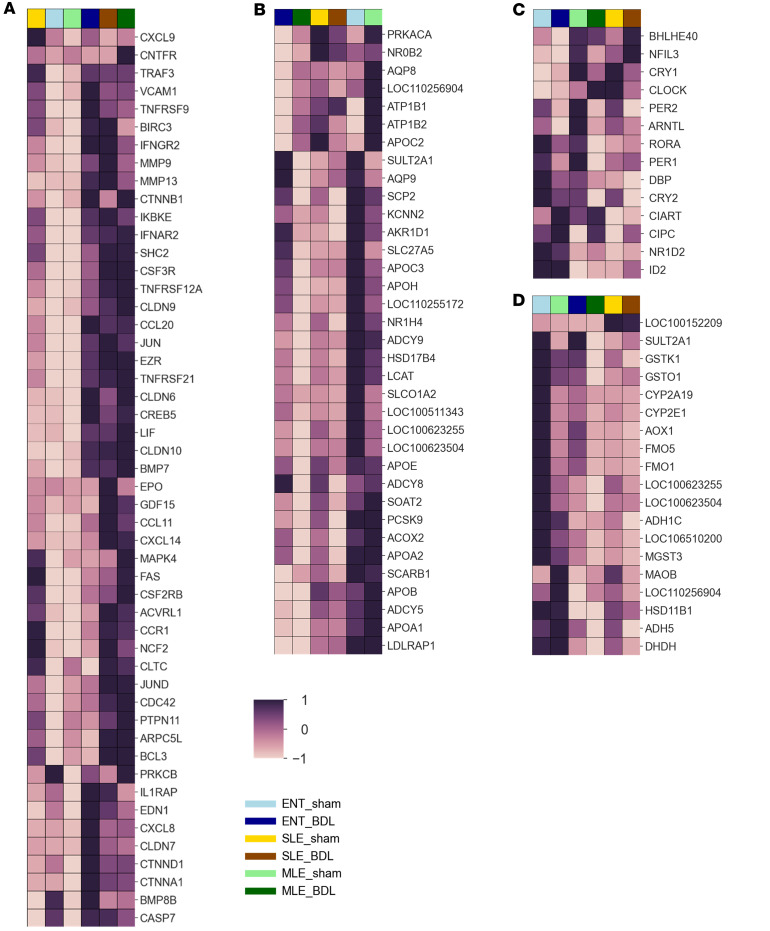
Gene expression of KEGG enriched pathways. (**A**–**D**) Heatmaps of significantly altered genes associated with KEGG pathways of inflammation (**A**), bile acid homeostasis (**B**), circadian rhythm (**C**), xenobiotics (**D**). *n* = 7–12/group.

Detailed examination of genes in significant pathways revealed that, in the xenobiotic pathway (Figure 6A) and bile acid pathways (Figure 6B), ENT and MLE groups clustered together, both sham and BDL, while SLE-sham and SLE-BDL clustered together. Circadian clock genes clustered by diet (Figure 6C). However, genes involved in inflammatory pathways (Figure 6D) clustered more prominently by sham versus BDL, independently of diet.

### Metabolomics identify reduced xenobiotic metabolites in PN-BDL groups compared with ENT-BDL.

**Figure 7:**
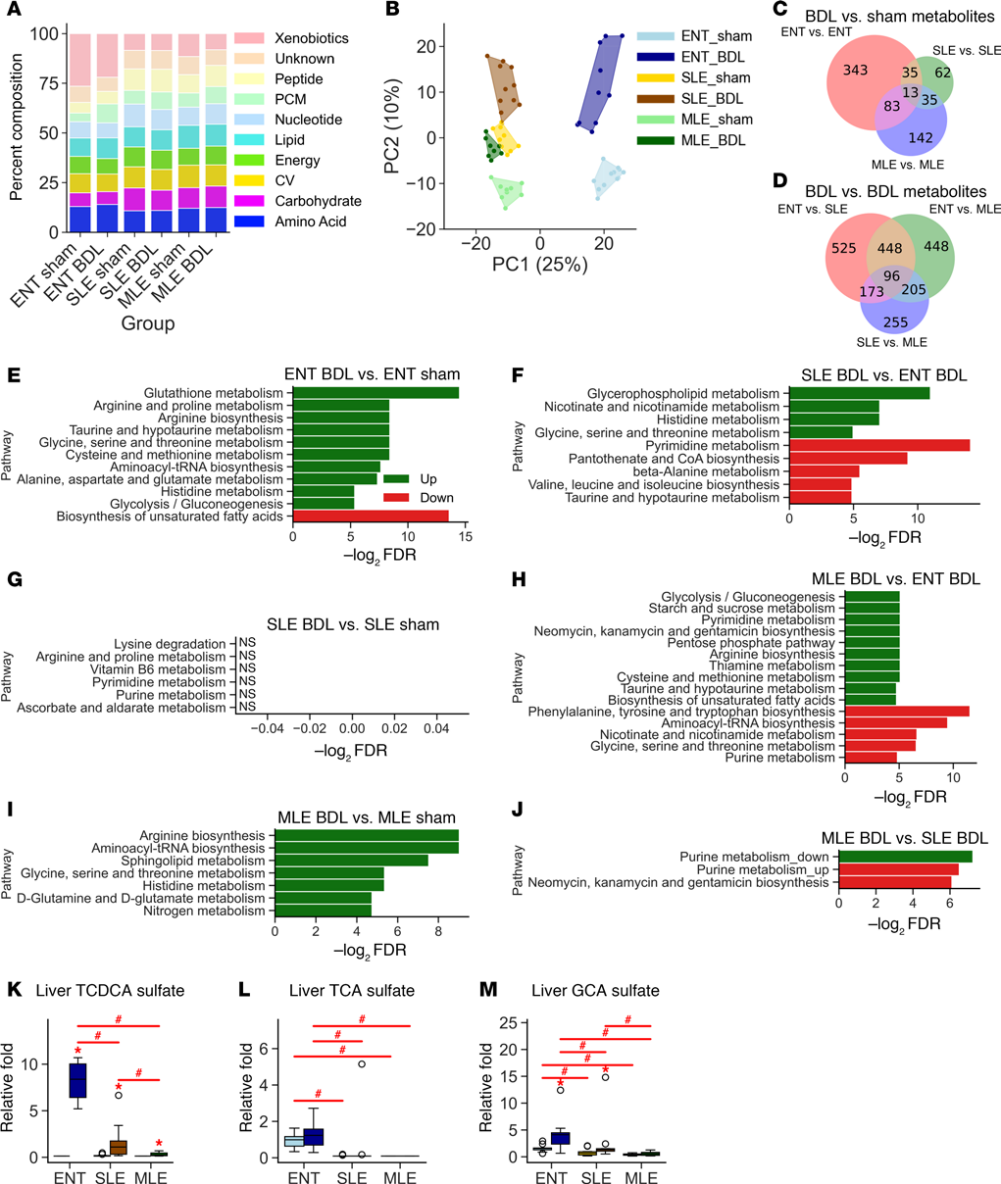
Metabolomics analysis confirms changes in xenobiotic metabolism and identifies amino acid metabolism pathways elevated in BDL in parenteral nutrition. (**A**) Composition of superpathways of metabolomic profiles. (**B**) Principle component analysis (PCA) of treatment groups. (**C** and **D**) Venn diagram of overlapping significantly altered metabolites (fold change > 2 and fdr < 0.05) for comparisons within-diet group (**C**) and between-diet (**D**) groups with BDL surgery. (**E**–**J**) Top 10 significantly upregulated (green) and downregulated (red) KEGG pathways for within-diet group (**E**, **G**, and **I**) (no significant groups detected in SLE-sham versus SLE-BDL comparison) and between-diet groups (**F**, **H**, and **J**) with BDL surgery. Individual metabolites selected from relevant pathways for (**K**–**M**) xenobiotic metabolism. Pathway significance was determined using Fisher’s exact test. Statistical significance for box plots was determined via 2-way ANOVA and Tukey’s post hoc comparison. *P < 0.05 from within-diet comparisons, ^#^*P* < 0.05 from within-surgery treatments. CV, Cofactors and Vitamins; GCA, glycocholic acid; PCM, Partially Characterized Molecules; TCA, taurocholic acid; TCDCA, taurochenodeoxycholic acid.

To confirm the findings of our RNA-Seq data, we conducted untargeted metabolomic profiling of liver samples. There was a large decrease in the total contribution of xenobiotic metabolites relative to the total percentage of all metabolites in the PN groups compared with the ENT groups (Figure 7A). Similar to our RNA-Seq data, PCA analysis showed a clear separation between ENT and PN groups. However, unlike our RNA-Seq data, the PN group clustering was closer by diet than BDL versus sham status (Figure 7B). The total number of significantly different metabolites within each diet was greatest in the ENT-BDL versus ENT-sham comparison (343 metabolites) and considerably lower in the SLE-BDL versus SLE-sham comparison (62 metabolites), with the MLE group comparison falling in between (Figure 7C). We observed more significantly different metabolites when comparing the BDL groups by different diet. The greatest number of differences was from the ENT-BDL versus SLE-BDL comparison (525 metabolites) (Figure 7D). The SLE-BDL versus MLE-BDL comparison yielded the lowest number of significantly different metabolites (255 metabolites). We then examined the enriched KEGG pathways of our treatment comparisons, and in general, there were much fewer significantly identified pathways as compared with our RNA-Seq analysis, given the much lower number of metabolites compared with genes. Within-diet comparison (Figure 7, E, G, and I) metabolites from arginine biosynthesis, histidine metabolism, and glycine, serine, and threonine metabolism pathways were upregulated in the ENT- and MLE-BDL groups. There were no significant differences within diet pathways in the SLE-BDL versus SLE-BDL comparisons. Surprisingly, there were no shared pathways among the BDL comparison between diets (Figure 7, F, H, and J). The higher number of significant differences were found in the MLE-BDL versus ENT-BDL comparisons, with more pathways related to amnio acid and energy metabolism being upregulated. Specifically, fatty acid composition was particularly affected by diet (Supplemental Figure 2, A–C). The MLE-BDL and MLE-sham groups had significantly higher DHA concentrations than either ENT or SLE groups. Contrary to what we expected to observe, both arachidonic acid and DHA were elevated in the ENT-BDL group compared with the ENT-sham group. However, when comparing the ratio of total omega-3 to omega-6 fatty acids, pigs within diet groups did not differ by sham versus BDL surgeries. Bile acid metabolites from xenobiotic metabolism pathways were altered. Sulfated-taurine conjugates of bile acids (Figure 7, K–M) were significantly elevated in the ENT-BDL group compared with SLE-BDL and MLE-BDL. Overall, the PN groups showed suppressed sulfation of bile acids in both sham and BDL groups. Lastly, we examined metabolites associated with oxidative stress through glutathione (Supplemental Figure 2, D–I). ENT-BDL had significantly lower oxidized glutathione (GSSG) and reduced glutathione (GSH). The PN groups also had lower GSSG compared with the ENT-sham group but were not different from ENT-BDL. However, despite there being a trend for lower concentrations, reduced glutathione was not different in the PN-BDL groups compared with their respective sham comparisons.

## Discussion

Previous results from our laboratory showed that MLE compared with SLE is protective against cholestatic liver injury in neonatal pigs given TPN (32, 39, 40). An observation in our previous study was that bile flow into the intestine was greater in pigs given MLE than those given SLE (32). This suggested that the benefit of MLE versus SLE was mediated by the maintenance of normal bile flow, thereby preventing cholestatic liver injury. We confirmed this in the current study as intestinal bile acid pool size in sham pigs administered PN with MLE was greater than those receiving SLE. In contrast, as we hypothesized, obstructing bile flow from the liver to the intestine with BDL eliminated the benefit of MLE to prevent cholestatic liver injury. Despite the failure to prevent cholestatic liver injury, the pigs with BDL receiving PN with MLE showed greater weight gain than both the enterally fed pigs and the PN pigs receiving SLE. This may be clinically important and consistent with our previous finding that MLE promotes more lean mass and less fat mass weight gain compared with SLE in neonatal pigs (41). Given that poor weight gain (i.e., failure to thrive) on enteral feeds is well documented in infants with BA (42–44), our results in neonatal pigs suggest that MLE may support better weight gain than SLE when PN is required to support infants with BA.

Clinically, the evidence of benefit of MLE use over SLE in PN has been equivocal. Some studies with small cohorts showed positive effects on markers of liver injury and cholestasis (45–47). However, meta-analysis and more recent clinical studies have cast doubt on the overall benefit of MLE over SLE (28, 48–50). The metabolic rational for the development of MLE was that a balanced fatty acid composition would be more beneficial than a high omega-6 enriched emulsion. The use of elevated omega-3 fatty acids, mainly DHA and EPA, in parenteral lipid emulsions has been reported to promote a less inflammatory environment than high omega-6 fatty acid emulsions (51, 52). However, in the current study, we did not see any difference in hepatic gene expression related to inflammation or liver injury between MLE compared with SLE or ENT pigs under BDL conditions. This was despite a strong proinflammatory gene expression signature for all the BDL compared with sham groups. We observed a decrease in both reduced and oxidized forms of glutathione in the PN groups. We postulate this high oxidative stress contributed to injury more than any benefit high omega-3 fatty acid may contribute.

The regulation of bile acid homeostasis via the gut/liver FXR/FGF19 signaling axis is an important feedback mechanism in preventing excessive bile acid accumulation in the healthy state (53). Obstructive cholestasis disrupts this mechanism by blocking the enterohepatic circulation of bile acids. In animal models, such as mice and rats, obstructive cholestasis through BDL causes a loss of intestinal FGF15 (FGR19 orthologue in rodents) production, leading to elevated bile acid production (54, 55). The evidence of disrupted FXR-FGF15/19 axis is usually measured by increased hepatic *CYP7A1* expression or by elevated serum levels of the oxysterol, C4. Unlike *FGF19* in humans, in rodents, *Fgf15* is only expressed in the intestine and not the liver (56). In humans, hepatic FGF19 expression is normally low or undetectable. In pigs, like in humans, FGF19 is present in the intestine and in the liver (40). However, when cholestatic conditions occur, hepatic FGF19 expression is increased (57–59). In infants with BA, FGF19 is elevated and CYP7A1 and C4 are reduced. Thus, infants with BA are still cholestatic despite the evidence of decreased bile acid synthesis through the classical pathway. Surprisingly, we found no increase in hepatic *FGF19* mRNA expression, and plasma FGF19 was barely detectable, despite the marked elevation of circulating and hepatic bile acids in our BDL pigs. Likewise, both *CYP7A1* mRNA and C4 were elevated consistently with low plasma FGF19 concentrations. This paradoxical finding of increased hepatic *FGF19* mRNA in the BA infant but not in the BDL pig is not entirely clear. We observed a large increase in the bile acid HCA, which is typically very low in humans. HCA is a muricholic acid, like the rodent β-muricholic acid (βMCA). Like βMCA, HCA is a poor agonist for FXR (60–62). In infants, the more potent FXR agonist CDCA is around > 60% in healthy infants and manages above 50% during cholestasis, though the concentration overall increases in the liver during BA (58). We postulate that the disproportionately high HCA and low CDCA composition that occurs in BDL pigs caused insufficient activation of the hepatic FXR/FGF19 axis.

The ENT-BDL pigs had lower hepatic and circulating bile acids levels compared with the BDL groups that were on TPN. Our results suggest that this lower bile acid accumulation in ENT versus TPN-BDL pigs was mediated by increased urinary bile acid clearance. Hydroxylation and conjugation of bile acids via hepatic xenobiotic metabolism facilitates their urinary excretion (63). We observed evidence of decreased hepatic xenobiotic metabolic genes in all BDL groups compared with the ENT-sham pigs, but the 2 PN BDL groups were even lower than the ENT-BDL, consistent with lower urinary output of bile acids. Likewise, our hepatic metabolomic analysis showed considerably lower sulfated bile acids in the 2 PN BDL groups compared with the ENT-BDL. Other groups have observed suppression of xenobiotic metabolism genes during administration of PN lipids in organoids (64) and mouse models (65). The mechanism for this suppression is not clear, but PN has been linked to altered hepatic circadian rhythm genes, which in turn could affect xenobiotic pathway genes (66, 67). We observed a modest change in hepatic circadian rhythm genes in PN versus ENT pigs that warrants further study.

Using pigs as a model organism offers several advantages over rodents, particularly in studies related to gastrointestinal (GI) function, liver function, and metabolism (68, 69). Furthermore, pigs have immune responses that are more comparable with humans, making them a valuable model for immunological studies (70). Our model uses neonatal pigs, which closely resemble human infants in terms of their developmental stage and metabolic profiles (71). However, there are some limitations of our model to note in the current study. Our model is representative of early-stage cholestasis at only 2 weeks of obstruction. This relatively short duration does not allow us to fully extrapolate the effects of different lipid emulsions on the long-term progression of liver disease to the end-stage liver failure that requires liver transplantation in humans. Chronic cholestasis involves a complex interplay of ongoing inflammation, fibrosis, and potentially the development of complications like portal hypertension, which may manifest differently over a longer period. Additionally, our approach of using BDL models 2 main consequences of BA: reduced bile flow to the gut and increased bile retention in the liver. While BDL effectively induces these key features and allows us to study the effect of lipid emulsions in this context, it’s important to acknowledge that it doesn’t fully recapitulate the initial and potentially more complex etiology of BA. For instance, BDL is a surgical procedure that causes an acute obstruction, whereas the pathogenesis of BA in infants is thought to involve a more gradual inflammatory and fibro-obliterative process of the extrahepatic bile ducts, potentially with a viral or genetic predisposition (72). These differing mechanisms could influence the downstream effects on the liver and other systems. Furthermore, in our study, the pigs receiving PN were maintained without any source of ENT feeds. This contrasts with the clinical reality for infants with BA, who, even when requiring PN support, will often still be fed enterally to the extent that they can tolerate. ENT feeding can have important effects on gut health, the microbiome, and the gut/liver axis, which could influence the response to different lipid emulsions (73). Therefore, our study, which used total PN, likely represents a more extreme clinical scenario, potentially reflecting infants with BA who have significant feeding intolerance or are in a phase where ENT feeds are severely limited.

The aim of this study was to examine whether the use of MLE is more effective than SLE on the prevention of liver injury in a model of neonatal obstructive cholestasis. The creation of hepatic biliary obstruction using BDL at birth led to a rapid induction of cholestasis based on serum chemistry and marked liver injury–evident histological features of ductular reaction and fibrosis within 14 days. The evidence of hepatobiliary injury and fibrosis was also observed in the upregulation of gene pathways involved inflammatory and fibrosis. Neonatal obstructive cholestasis was also marked by uncoupling of the enterohepatic FXR/FGF19 signaling and loss of negative feedback on hepatic bile acid synthesis via both the classis and alternative pathways. In this context of neonatal obstructive cholestasis, our findings suggest that the use of MLE does not provide sufficient hepatic protection against injury or inflammation during cholestasis compared with SLE but may confer a benefit of improved growth. Our findings should inform the clinical use of MLE versus SLE in infants diagnosed with BA who require PN support.

## Methods

### Sex as a biological variable.

Our study examined male and female animals, and similar results were obtained for both sexes.

### Animal and study design.

Sows of mixed Landrace and Hampshire breeds were purchased from a commercial farm in Texas (Real Hog Farms, Marion, Texas, USA). Pigs were delivered via cesarean section 1 day preterm (day 113 gestation) as described previously (40). Immediately following delivery, pigs were randomly assigned to either BDL or sham surgery. BDL surgery consisted of a ventral midline incision of the abdomen from the xyphoid process to the umbilicus. The cystic duct and common bile duct were both ligated. For sham surgery, the ventral midline incision was made, and the liver was manipulated briefly before closing. Subsequently, all pigs were then implanted with a jugular catheter. Within the sham and BDL groups, pigs were then randomly assigned to receive nutrition through either ENT feeding of a milk replacer formula — NutraStart Liqui-Wean (ENT-sham, ENT-BDL; Milk Specialties Global) — or PN containing either SLE, Intralipid (SLE-sham, SLE-BDL; Fresenius Kabi), or MLE SMOFlipid (MLE-sham, MLE-BDL; Fresenius Kabi). The pigs receiving ENT feeding were implanted with an orogastric tube. The ENT and TPN groups were calorically matched to achieve an intake of 195 kcal/(kg·d). The macronutrient intake during full PN intake was 25 g/(kg·d) glucose, 13 g/(kg·d) amino acids, 5.0 g/(kg·d) lipid. All pigs received TPN for the first 24 hours after delivery. Enterally fed pigs were gradually taken off of TPN, reaching full ENT feeds on day 7. Study was completed 14 days after delivery.

### Growth rate and tissue weights.

Pigs were weighed at birth and at time of tissue harvest on day 14 of life. To determine growth rates, pigs’ birth weight and final body weights were averaged over the period of the study and expressed in grams per kg·d. The liver and the small intestine were collected at the time of sacrifice. The small intestines are defined for this study as the ligament of Treitz to the cecum. The intestines were washed with normal saline prior to weighing to remove any excess contents.

### Tissue staining and quantification.

All histological studies utilized formalin-fixed paraffin-embedded samples. Liver tissue from the left lobe was dissected, fixed in formalin for 24 hours, and subsequently transferred to 70% ethanol. These sample cassettes were then submitted to the Cellular and Molecular Morphology Core of the Texas Medical Center’s Digestive Disease Center for slide mounting and IHC. Cholangiocytes were stained using anti-panCK I/II (MA5-13156, Thermo Fisher Scientific). To assess liver collagen deposition, slides were stained with Sirius red following an established protocol (74). For image quantification, slides were scanned at 20× magnification using the Zeiss AxiosScan.Z1. The Zen 3.3 software package (Zeiss) was used to open slide images, select 6 identical-sized random square fields (9 mm^2^) within the slide image, and convert these fields to PNG files for further analysis using ImageJ (NIH). For the panCK analysis, color deconvolution was applied to each image field to yield DAB (panCK). Color thresholds were adjusted to eliminate background noise. Prior to percent area counting, the particle size was set to > 100 pixels^2^ to exclude artifacts. For Sirius red staining, the image was divided into red, green, and blue channels. The red channel, which provided the greatest contrast between collagen staining and the background, was used for quantification. Thresholding and particle size exclusion were performed as above to remove background and artifacts. The average area coverage per field from each slide was used for each sample in the statistical analysis.

### qPCR.

Liver tissue was homogenized in Trizol (Thermo Fisher Scientific) and total RNA isolated using RNAeasy Spin columns (Qiagen). A total of 1 μg total RNA was converted to cDNA using the High Capacity cDNA Synthesis Kit (Qiagen). For each gene (Supplemental Table 1), 10 ng of cDNA was used in a qPCR reaction with Power Up Sybr Green (Applied BioSciences) on a Biorad CFX-96 instrument. Relative fold was calculated using the 2^ΔΔCt^ method, and *ACTB* primers were used as the normalization gene as done previously (40).

### Bile acid and oxysterol composition analysis.

Bile acids were prepared from plasma samples as described previously (32). For oxysterol analysis, a mixture was prepared consisting of 200 μL of plasma, 200 μL of methanol, 400 μL of dichloromethane, and 100 μL of liquid chromatography–mass spectrometry–grade (LC-MS–grade) water. To this mixture, 50 μL of an internal standard mixture solution (containing 5.81 μmol cholest-5-ene-3β,22(S)-diol-d7 (Avanti Polar Lipids, Alabaster, Al) and 5.71 μM cholest-5-en-3β,7α-diol-d7 (Avanti Polar Lipids) was added. The sample mixture was then vortexed for 20 seconds and centrifuged at 4500 x g at 8°C for 10 minutes. The lower dichloromethane layer was collected separately and dried under nitrogen. The dried dichloromethane phase was reconstituted in 500 μL of methanol and 1.5 mL of LC-MS–grade water containing 0.1% formic acid, and then vortexed. Oasis HLB SPE cartridges (Waters Corp.) were primed by passing 800 μL of methanol and 600 μL of water with 0.1% formic acid. The sample was loaded onto the column and allowed to pass through by gravity, with the eluent being discarded. The samples were then washed with 600 μL of LC-MS water containing 0.1% formic acid, followed by a wash with 600 μL of hexane. The oxysterol fraction was then eluted with butyl acetate from the SPE cartridge and dried under vacuum. Finally, the residue was dissolved in 100 μL of buffer (consisting of 70 μL of MeOH [5mM ammonium acetate] and 30 μL water [5mM ammonium acetate]). Quantitation was performed using LC-MS/MS as described previously (32).

### Serum chemistry.

Blood samples were collected into serum tubes and K2 EDTA tubes and processed to yield serum and plasma, respectively. Serum was analyzed on a Roche-Cobas 6000 analyzer as described in detail previously (75). Plasma was used to analyze all other compounds as described.

### Total bile acid quantitation.

To determine total bile acids in plasma, a commercially available kit was used according to manufacturer’s protocol (GWB-BQK087; GenWay Biotech) as described previously (40). For liver, bile, and small intestine samples, the sample was homogenized in ethanol, and the supernatant used for bile acid quantitation. Bile acid pools were calculated as described previously (40).

### RNA-Seq analysis.

RNA was isolated from liver samples and provided to Novogene for RNA-Seq pipeline. Using their PE150 sequencing strategy, 20 million raw paired end reads were generated and matched to sus scrofa assembly GCF_000003025.6_Sscrofa11.1. Briefly, RNA quality was assessed using agarose gel electrophoresis and NanoPhotometer followed by quantification and integrity analysis with the Agilent Bioanalyzer 2100 system. Total RNA (1 μg) was used for library preparation with NEBNext UltraTM RNA Library Prep Kit for Illumina followed by purification and size selection. Libraries were sequenced on an Illumina platform, and clean data were obtained through quality control steps including processing through fastp to remove reads containing adapters, poly-N sequences, and low quality reads. Reads were mapped to a reference genome using HISAT2, and novel genes were predicted with Stringtie and gffcompare. Featurecounts was used to quantify gene expression (RPKM), and differential expression analysis was performed using DESeq2 (>2-fold expression difference and False Discovery Rate [FDR] < 0.05). Finally, enrichment analysis for KEGG pathways was performed using clusterProfiler R to identify significantly (adjusted *P* > 0.05) enriched biological functions.

### Metabolomics analysis.

Frozen liver samples were analyzed by Metabolon using their proprietary platform (76). Briefly, upon arrival, samples are stored at –80°C and assigned unique identifiers for tracking. For sample preparation, recovery standards were added to samples,and then samples were precipitated with methanol under shaking to remove protein and to free bound metabolites. The resulting extract was then divided for analysis using 4 different ultraperformance LC–tandem MS (UPLC-MS/MS) methods using a Waters ACQUITY UPLC and a Thermo Fisher Scientific Q-Exactive high resolution/accurate mass spectrometer interfaced with a heated electrospray ionization (HESI-II) source and Orbitrap mass analyzer operated at 35,000 mass resolution. To ensure data quality, a combination of pooled matrix samples, process blanks, and QC standards are included throughout the analysis. Data analysis was performed using peak identification, quality control procedures, and metabolite quantification using peak AUC with proprietary Metabolon software. Statistical analysis was performed by Metabolon using a 2-way ANOVA with FDR (*q* value) > 0.05 used to define significant interactions between groups. For pathway analysis, significant metabolites were analyzed using the web-based multi-omics software suite, MetaboAnalyst (77). Pathways that were enriched with > 2-fold difference between groups and *q* < 0.05 were included as significantly enriched pathways.

### FGF-19 assay.

A commercially available kit (ELP-FGF19-1, Raybiotech) was used according to manufacturer’s specification on plasma samples as described previously (78).

### Statistics.

All values are graphically presented as mean ± SEM, and the individual pig values are included in figures. The final day samples were analyzed by 2-way ANOVA and interactions between diet (ENT, SLE, MLE) and surgery (sham, BDL) were tested for significance using Tukey’s post hoc analysis. Statistical analyses were performed using Minitab and GraphPad software packages. For data that were not normally distributed or were of equal variance, log transformations were performed prior to statistical analysis.

### Study approval.

All experiments were approved by the Animal Care and Use Committee of Baylor College of Medicine and conducted in accordance with the *Guide for the Care and Use of Laboratory Animals* (National Academies Press, 2011).

### Data availability.

All data from Figures 1–4, and 7 are included in the Supplementary file Supporting Data File. RNA-Seq data (Figures 5 and 6) are available for download on the NCBI GEO database website at GSE281589.

## Author contributions

GG and DB conceptualized the work; GG, CV, VMH, and BS performed experiments and collected the samples; GG and BS analyzed the data; GG wrote the first draft of the manuscript; GG, CV, VMH, BS, and DB edited the manuscript. All authors reviewed and approved the manuscript.

## Supplementary Material

Supplemental data

Supporting data values

## Version 1 04/17/2025

In-Press Preview

## Version 2 05/22/2025

Electronic publication
