# Supplementary material for: Multicomponent parenteral lipid emulsions do not prevent liver injury in neonatal pigs with obstructive cholestasis
Source: JCI Insight. 2025 Apr 17;10(10):e189196. doi: 10.1172/jci.insight.189196 (PMC12128955; doi:10.1172/jci.insight.189196)
Supplement: Supplemental data [file jciinsight-10-189196-s192.pdf]

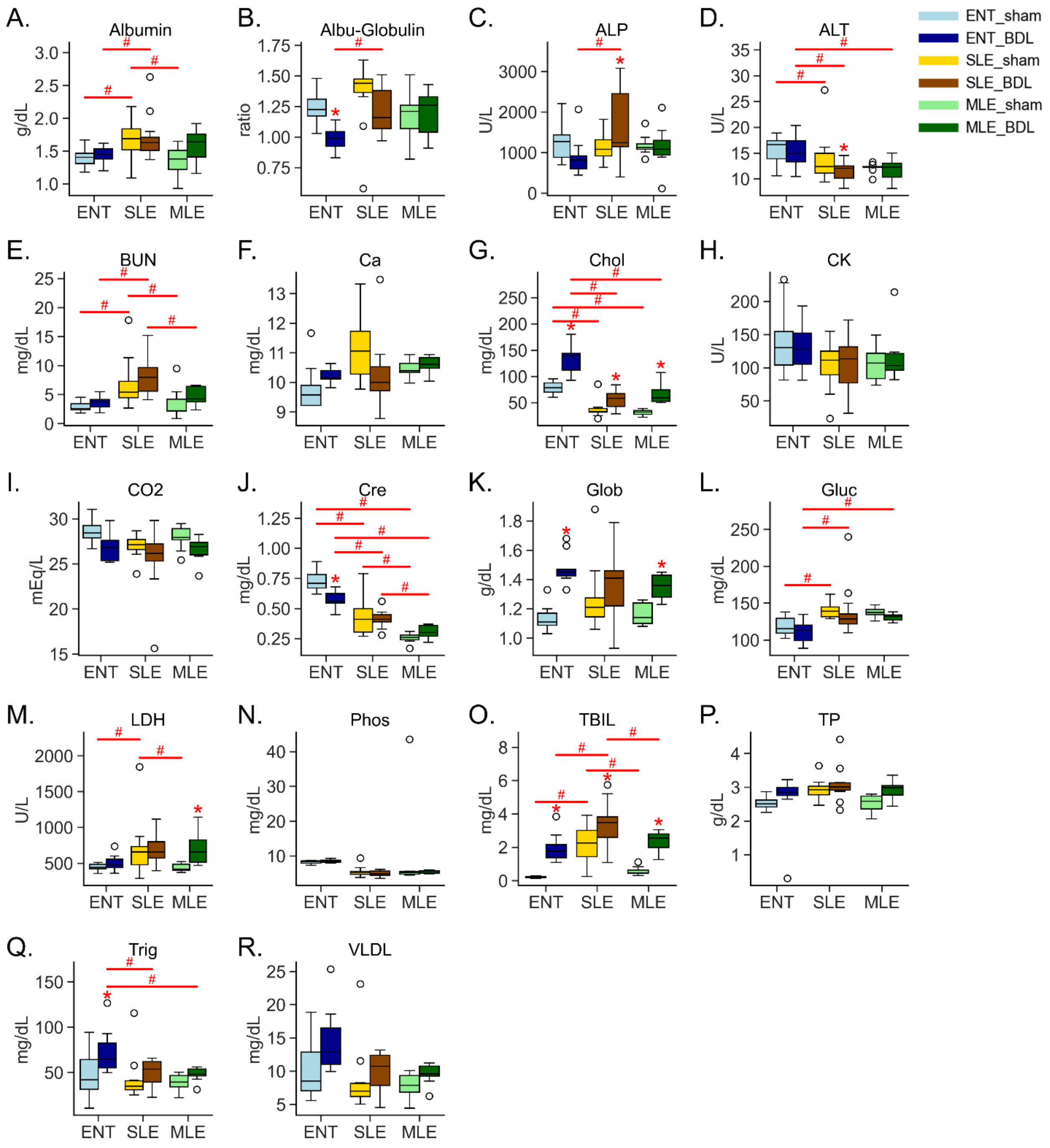

Supplementary Figure 1. Serum chemistry panel. Statistical significance for box plots was determined via two-way ANOVA and Tukey's post hoc comparison. Values with * are significantly different ($P < 0.05$) from within Diet comparisons and values with # are significantly different ($P < 0.05$) from within Surgery treatments. Box plots lines represent quartiles, whiskers represent largest value within 1.5x interquartile range, open circles represent outliers. n = 9-12/group. Alkaline phosphatase, ALP; alanine aminotransferase, ALT; blood urea

nitrogen, BUN; calcium, Ca; total cholesterol, Chol; creatine kinase, CK; carbon dioxide, CO_2; creatinine, Cre; globulin, Glob; glucose, Gluc; lactate dehydrogenase, LDH; phosphorous, Phos; total bilirubin, TBIL; total protein, TP; triglyceride, Trig; very low density lipoprotein, VLDL.

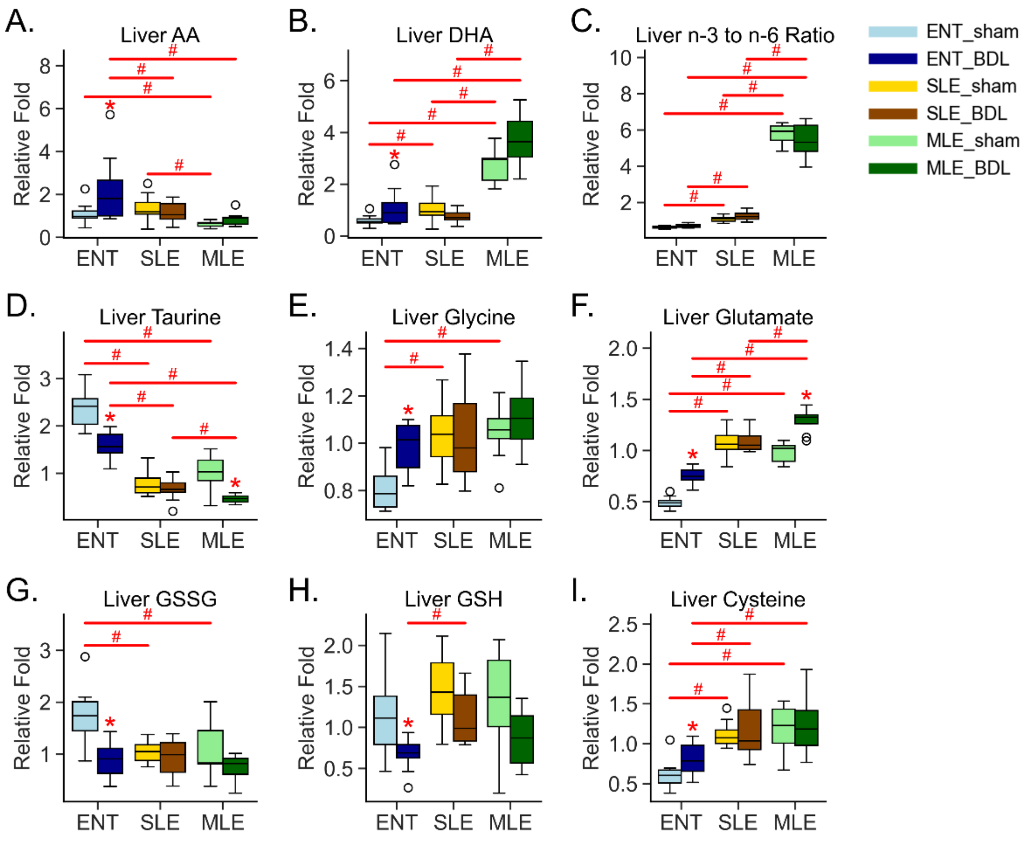

Supplementary Figure 2. Metabolomic data for fatty acid and glutathione synthesis. Individual metabolites selected from relevant pathways for (A-C) fatty acid composition and (D-I) amino acid and glutathione metabolism. Statistical significance for box plots was determined via two-way ANOVA and Tukey's post hoc comparison. Values with * are significantly different ($P < 0.05$) from within Diet comparisons and values with # are significantly different ($P < 0.05$) from within Surgery treatments. Arachidonic acid, AA; docosahexaenoic acid, DHA; oxidized glutathione, GSSG; reduced glutathione, GSH.

Supplementary Table 1. PCR Primers

Gene Name	Accession #	Forward	Reverse
Cyp7a1	NM_001005352.3	GAAAGAGAGACCACATCTCGG	GAATGGTGTTGGCTTGCGAT
Sox9	NM_213843	GAAAGTCGGTGAAGAACGGC	TTGGGAGAGATGTGCGTCTG
Mmp7	NM_001348795.1	AACACTGGTCTGATGGTGGC	CAGACGAATGAGCCAGACCT
Tgfb	NM_214015.2	ACCTGCAAGACCATCGACAT	GACAGAATCTGGCCGCGAAT
